# Human Cytochrome P450 2W1 Is Not Expressed in Adrenal Cortex and Is Only Rarely Expressed in Adrenocortical Carcinomas

**DOI:** 10.1371/journal.pone.0162379

**Published:** 2016-09-06

**Authors:** Paola Nolé, Britt Duijndam, Adam Stenman, C. Christofer Juhlin, Mikael Kozyra, Catharina Larsson, Magnus Ingelman-Sundberg, Inger Johansson

**Affiliations:** 1 Department of Physiology and Pharmacology, Karolinska Institutet, Stockholm, Sweden; 2 Department of Oncology-Pathology, Karolinska Institutet, and Cancer Center Karolinska, Karolinska University Hospital, Stockholm, Sweden; University of South Alabama Mitchell Cancer Institute, UNITED STATES

## Abstract

Human cytochome P450 2W1 (CYP2W1) enzyme is expressed in fetal colon and in colon tumors. The level of expression is higher in colon metastases than in the parent tumors and the enzyme is a possible drug target for treatment of colorectal cancer, as demonstrated in mouse xenograft studies. A previous study published in this journal reported that CYP2W1 is highly expressed in normal and transformed adrenal tissue. However, adrenal expression of CYP2W1 protein was not seen in previous studies in our research group. To clarify this inconsistency, we have used qRT-PCR and Western blotting with CYP2W1-specific antibodies to probe a panel of 27 adrenocortical carcinomas and 35 normal adrenal cortex samples. *CYP2W1* mRNA expression is seen in all samples. However, significant CYP2W1 protein expression was found in only one tumor sample (a testosterone-producing adrenocortical carcinoma) and not in any normal tissue. Differences in the specificity of the CYP2W1 antibodies used in the two studies may explain the apparent discrepancy. We conclude that normal adrenal tissue lacks P450 2W1 enzyme expression; also, adrenocortical carcinomas generally do not express the enzyme. This information thus underline the colon cancer specificity of CYP2W1 enzyme expression and has implications for the development of anti-colon cancer therapies based on CYP2W1 as a drug target, since 2W1-dependent bioactivation of prodrugs for CYP2W1 will not take place in normal adrenal tissue or other non-transformed tissues.

## Introduction

We have reported that cytochrome P450 2W1 (CYP2W1) is expressed in about 30% of colorectal carcinoma (CRC) samples and 50% of liver metastases of CRC, but not in normal (non-transformed) adult intestine [1], [2]. In contrast, specific expression of CYP2W1 in the small intestine and colon is seen in fetal mice and in human fetuses (Choong et al. 2015 [3]). Expression begins at an early stage in the embryo and is silenced shortly after birth by increased methylation of CpG-rich regions in the murine and human *CYP2W1* genes [3]. In CRC, CYP2W1 expression correlates with the extent of demethylation of these CpG islands, which are located mainly near the exon1-intron 1 boundary [2].

CYP2W1 expression in colon carcinoma is a prognostic factor: high expression is associated with poor survival [4], [5]. Since almost half of CRC metastases express CYP2W1, this enzyme is a possible target for treatment of CRC liver metastasis [6]. We have shown, using both a cell culture model and a mouse xenograft model (CYP2W1-expressing human colon carcinoma cells), that duocarmycin (chloromethylindolines) prodrugs can be converted to cytotoxic products by CYP2W1 [7]. The cytotoxicity of these prodrugs is mediated by a CYP2W1-dependent oxidation followed by spirocyclization and production of an N3-adenine covalent adduct [7,8]. By the administration of the prodrugs, the growth of the human CYP2W1 colon cancer cells in the mouse xenografts is arrested as seen from the lack of further increase of the xenograft size, whereas no effect is seen on growth of xenografts without CYP2W1. Furthermore, toxicity was not observed in the control mice, following drug administration. Several different compounds have been suggested as endogenous substrates, among them retinoic acid, see Guo et al., 2016 [9].

Recently, Ronchi *et al*. [10] reported high CYP2W1 protein expression in adrenocortical carcinomas (ACC), as well as in normal adrenal tissue. The major aim of that study was to assess the prognostic role of CYP2W1 in ACCs. In total, 239 ACCs were analyzed using CYP2W1 immunohistochemistry and the results compared with those from adrenocortical adenomas, normal adrenals, other normal non-adrenal tissues, and several non-adrenocortical cancer forms. The authors concluded that CYP2W1 immunoreactivity is high in 65% of normal adrenal glands and in 50% of ACCs.

These results contradict a previous study that failed to identify significant expression of CYP2W1 protein in any non-transformed adult human tissue and in ACC [1] as well as our recent preliminary studies. Expression of CYP2W1 in any normal tissue carries the risk of toxic side effects of CYP2W1-targeted cancer therapy. We found it of high importance to test the findings by Ronchi *et al*. [10], using additional patient and control cohorts and another antibody towards CYP2W1, one previously shown to be specific for the CYP2W1 enzyme [1], [2]. Our data indicate that significant CYP2W1 protein expression occurs neither in normal adrenal cortex tissue (nADR) nor in ACC (with one exception); the results obtained by Ronchi *et al*. [10] may have been caused by antibody cross-reactivity.

## Material and Methods

### Patients and samples

Fresh frozen samples of ACCs (n = 27), nADR (n = 35), and a matched normal adrenal cortex tissue sample corresponding to sample Ca 29 (nADR-Ca 29) were obtained from the Karolinska University Hospital, Stockholm, Sweden. The nADR were adjacent normal tissue from surgery of ACC, adrenal cortical adenoma, pheochromocytoma or renal cell carcinoma. The ACC samples usually presented 70–90% tumor cell content and the normal samples exhibited 60–100% normal adrenocortical cells. Before surgical resection, written informed consent was obtained from all patients, according to protocols approved by the local Ethics review board at Karolinska Institutet, Stockholm. Clinical data are shown in S1 Table. As indicated in this table, subsets of the ACC samples have been included in previous publications [1], 10, [11].

Sample from HEK293-2W1 cell line was used as positive standard for the Western blot analysis of CYP2W1 and the corresponding cell line lacking CYP2W1 (mock cells) was used as negative control (ref Sheldrake = 8).

### Quantitative real-time PCR (qRT-PCR)

Total RNA was extracted using the *mir*Vana miRNA Isolation Kit (Ambion, Austin, TX, USA) and subsequently analyzed in an Agilent 2100 Bioanalyzer (Agilent, Santa Clara, CA, USA). RNA preparations from all samples maintained high quality and showed a mean RIN value of 7.85 ± 0.94, as previously reported [12]. RNA was prepared from 27 ACC samples, 11 nADR samples and normal adrenal cortex from the Ca29 patient (nADR-Ca29). cDNA was synthesized using SuperScript^™^ III Reverse Transcriptase (Invitrogen, Life Technologies, Carlsbad, CA, USA) and aliquots corresponding to 20 ng total RNA were subjected to expression analysis. Taqman gene expression assays (Life Technologies, Carlsbad, CA, USA) were used for *CYP2W1* (Hs00908623_m1) and the housekeeping gene TATA-binding protein (*TBP*, Hs00427620_m1). The average *TBP* expression was identical for ACC and nADR samples. Relative *CYP2W1* mRNA expression levels compared to expression in HepG2 cells were calculated from threshold cycle values and normalized to the housekeeping gene using the 2^^ΔCt^ method [13].

### Western blot analysis

Aliquots of tissue samples were homogenized using a Bullet Blender Homogenizer (Next Advance Inc., NY, USA) in three volumes of buffer containing protease inhibitor (Complete Tablets, Roche Diagnostics, Mannheim, Germany) and subsequently centrifuged for 10 min at 800 x g. Aliquots of the supernatants (corresponding to 25 μg protein) were subjected to SDS-PAGE and Western blot analysis according to Karlgren *et al*. 2006 [1] with minor modifications. AmershamProtran membrane (GE Healthcare Life Sciences, Little Chalfont, Buckinghamshire, UK) was used and visualization was performed using the Super Signal West Femto Chemiluminescent Substrate (Thermo Fisher Scientific Inc., Waltham, MA USA). The polyclonal antibodies 852 used were raised in rabbit against a 15 amino acid residue sequence from the C-terminal end of human CYP2W1 [1] and have been validated for specificity in previous publications [1], [14] (Antibody B in S1–S3 Figs). For comparison, the CYP2W1 antibody #1, used for IHC by Ronchi *et al*. [10], a polyclonal antibody against the N-Terminal region of CYP2W1 (PA5-14900, Thermo Fisher Scientific Inc., epitope: AQDPSPAARWPP) was also evaluated (antibody A in S1–S3 Figs). In addition further comparative analyses were carried out using a novel a mouse monoclonal antibody raised against amino acids 121–295 of CYP2W1 (sc-374426, Santa Cruz Biotechnology Inc., Dallas Texas, USA) (antibody C in S1–S3 Figs). All samples were analyzed in at least two independent Western blot runs (data shown in Additional supplemental information).

The tissue blot (Cat no 1522) was from BioCat GmbH, Heidelberg, Germany.

### Statistics

Statistical analysis was performed using Student’s t-test, GraphPad Prism v.5.0, GraphPad Software, La Jolla, CA, USA.

## Results

**Fig 1:**
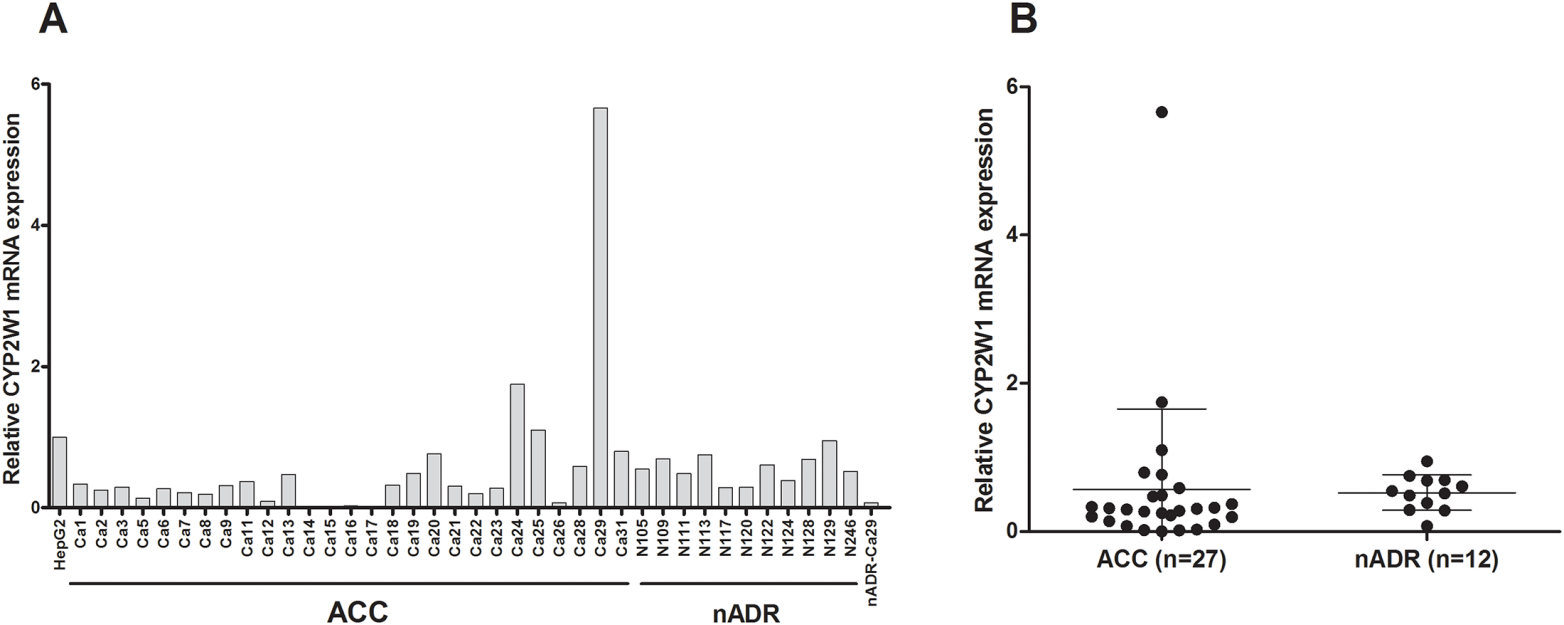
*CYP2W1* mRNA expression in adrenocortical carcinoma and normal adrenal cortex tissue. A. *CYP2W1* mRNA expression in adrenocortical carcinoma (Ca) and normal adrenal cortex tissue (N) compared to the expression in HepG2 cells. nADR-Ca 29 represents normal adrenal cortex from the patient Ca 29. B. The scatterplot shows mean ± SD of *CYP2W1* mRNA expression in ACC and nADR samples, p = 0.89.

*CYP2W1* mRNA expression was examined in 27 ACC samples and in 12 nADR. In general, ACC as well as nADR showed detectable but low *CYP2W1* mRNA expression levels, compared to the HepG2 reference (Fig 1). Ct-values were 21.8–37.4 for the ACC samples and 24.4–26.4 for the nADR samples. Relative *CYP2W1* mRNA expression levels compared to expression in HepG2 cells were calculated and the relative expression was found to be similar in nADR (mean 0.52, median 0.55) compared in ACC (mean 0.57, median 0.30), with no statistically significant different between groups (p-value 0.89). One tumor sample only (Ca 29) displayed very high *CYP2W1* mRNA expression, relative level 5.66 as compared to HepG2 cells. Normal adrenal cortex tissue from the same patient (nADR-Ca 29) expressed very low levels of *CYP2W1* mRNA (0.08) (Fig 1).

**Fig 2:**
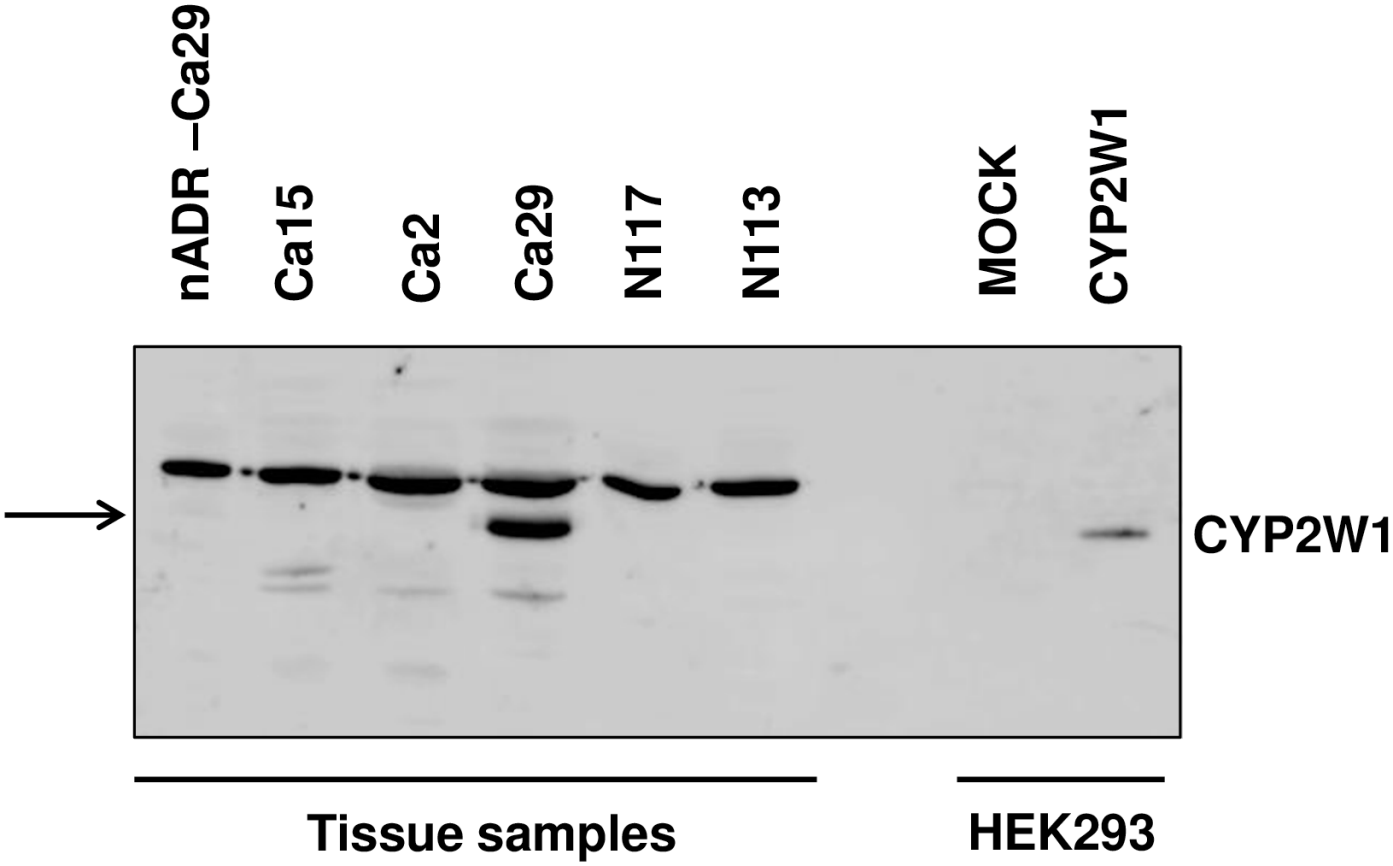
Western blot analysis of CYP2W1 expression in adrenocortical carcinoma (Ca) and normal adrenal cortex tissue (N) using the antibody 852 against a 15-residue sequence in the C-terminal end of the human CYP2W1 (Karlgren *et al*. 2006 [1]). A sample of HEK293 cells with stable expression of CYP2W1 was used as positive control and the corresponding Mock cells as negative control. nADR-Ca 29 represents normal adrenal cortex from the patient Ca 29. Arrow indicates the location of CYP2W1.

By Western blot analysis using the 852 antibody raised against the C-terminal of the CYP2W1 protein [1], [14], only one tumor sample showed significant CYP2W1 protein levels (Ca 29) (Fig 2). None of the 35 nADR samples showed detectable CYP2W1 apoprotein, but a weak band was visible in the non-tumor tissue from patient Ca 29. This might be explained either by tissue contamination from the high CYP2W1 protein expression in the tumor sample or by induction of expression by the tumor (Fig 2).

CYP2W1 protein expression did not correlate to the corresponding mRNA expression, except for tumor Ca 29. In order to verify the sensitivity of the antibody in the Western blot analysis used for the CYP2W1 protein, a series of dilutions of tumor sample Ca 29 were analyzed. As little as 0.78 μg protein from tumor Ca 29 resulted in a positive signal for CYP2W1, less than 1/30 of the amount of protein that was used for Western blot screening of the tumor and control samples (data shown in Additional supplemental information).

Ronchi *et al*. [10] used a commercial antibody from Thermo Fisher Scientific against the N-Terminal region of CYP2W1. In order to find the cause for the discrepancy between our results and those of Ronchi *et al*., we performed additional Western blot analyses using three different antibodies towards human CYP2W1 and samples encompassing negative controls, positive controls with recombinantly expressed CYP2W1, normal tissue from adrenal glands, a colon tumor as well as a blot containing proteins from 9 different human tissues (S1–S3 Figs). As shown in S1–S3 Figs, the Thermo Fisher Scientific antibody did not detect the CYP2W1 protein specifically; rather, several other protein bands were detected and on the tissue blot it recognized more than 10 different protein bands in each of the different normal tissue samples. By contrast, the monoclonal CYPW1 antibody and our 852 antibody recognized positive controls and CYP2W1 in colon cancer and in ACC and gave just a few bands in the tissue blot. These results indicate that the Thermo Fisher Scientific antibody cannot discriminate between CYP2W1 protein and other proteins in nADR and ACC. S1 and S2 Figs reveal that the specificity of the monoclonal mouse antibody from Santa Cruz is high and very similar to that of the 852 antibody,

## Discussion

Only one of the ACC samples, Ca 29, showed a very high level of CYP2W1 expression for both mRNA and protein level. Interestingly, this tumor was also one of three testosterone-secreting tumors in the set of samples. Ronchi *et al*. [10] noticed a correlation between hormone secretion from a tumor and the level of CYP2W1 expression, whereas we did not observe such a correlation. We saw no significant CYP2W1 protein expression in any of the other 26 tumors, nor in any of the samples representing nADR. This is in contrast to the results of Ronchi *et al*. [10], who reported CYP2W1 expression in normal adrenal glands. These apparently conflicting results can be resolved by recognizing the different antibodies used. When we tested the CYP2W1 antibody from Thermo Fisher Scientific, the antibody recognized several proteins with mobility similar to CYP2W1. The specificity of the 852 antibody is confirmed by the similar results obtained with the novel CYP2W1 monoclonal Santa Cruz antibody mainly identifying CYP2W1 but also some other protein. Support to our conclusion of a specific CYP2W1 protein expression only in fetal colon (see above) is found in the Human Gene Database “GeneCards” (www.genecards.org). Here, expression of CYP2W1 mRNA is found in several tissues, but the CYP2W1 protein is only detectable in fetal colon. Extrapolation of antibody specificity between Western blot analyses and IHC is always difficult since the structures of the exposed proteins will be different. It is clear that Western blotting with the additional discrimination with respect to molecular weight provides a higher specificity and it might be that the 852 Ab can cross react with contaminating proteins in the IHC in the experiments done by Ronchi *et al*.

Regarding mRNA expression, we identified significant expression of the *CYP2W1* gene in most of the samples, including the nADR samples, in line with the data of Ronchi *et al*. [10]. However, expression levels of *CYP2W1* mRNA and the corresponding protein levels were discrepant in both ACC and nADR samples. ACC sample Ca 24 showed a *CYP2W1* mRNA expression level of 40% compared to ACC sample Ca 29, but still undetectable levels of CYP2W1 protein. The sensitivity of the immunoblots allowed detection of at least 3% of the level in the high CYP2W1-expressing tumor. This confirms that significant expression of CYP2W1 protein was present in only one tumor sample, and that all other samples lacked detectable expression of this enzyme.

The reason for the lack of correlation between *CYP2W1* mRNA and protein expression is not known, but proteomic and transcriptomic analyses of samples are often highly discrepant, due to translational and post-translational control of protein expression.

The CYP2W1 protein is expressed in fetal intestine [3] but is rapidly down-regulated by gene hypermethylation after birth. Significant expression of this protein has not been identified in any normal adult tissue. In patients with liver metastases of CRC, almost half of the tumors express CYP2W1 protein. This fact, together with the lack of significant expression in any normal tissue, make CYP2W1-dependent duocarmycin prodrugs attractive candidates for future treatment of this group of patients [7].

In summary, significant expression of CYP2W1 should be expected only in rare cases of ACC. Lack of CYP2W1 protein expression in any normal adult tissue, including normal adrenal cortex, makes the enzyme a promising target for future colon cancer specific therapy.

## Supporting Information

S1 Fig Comparison of three different CYP2W1 antibodies. Comparison of three different CYP2W1 antibodies using WB filters with 800 x g supernatant from HEK293 cells expressing CYP2W1 and the corresponding cells without CYP2W1 expression (HEK-Mock). Antibodies used: A Thermo Fisher Scientific PA5-14900, B Our C-term ‘852’, C SantaCruz monoclonal sc-374426. WB filter 1 was first incubated with antibody A using goat anti-rabbit IgG-HRP for visulization and subsequently with antibody B using the same secondary antibody. WB filter 2 was fist incubated with antibody C using goat anti-mouse IgG-HRP for visualization and subsequently with antibody B using goat anti-rabbit IgG-HRP. The indicated time corresponf to exposure time.
(TIF)

S2 Fig Comparison of three different CYP2W1 antibodies. Thermo Fisher Scientific PA5-14900 antibody A, Our C-term ‘852’ (antibody B), C SantaCruz monoclonal sc-374426 (antibody C). Four identical WB filters were prepared with 800 x g supernatant from HEK293 cells expressing CYP2W1 (diluted sample) and the corresponding cells without CYP2W1 expression (HEK-Mock), normal adrenal cortex, nADR129, (25 μg protein) and a colon cancer sample with previously known expression of CYP2W1 (25 μg protein). **S2A Fig**: The left part of filter 3 was incubated with the Thermo Fisher antibody and the right part with our 852 antibody. The former generated a lot of bands also having mobilities similar to CYP2W1, whereas the 852 antibody visualized CYP2W1 only in the colon cancer sample and after longer exposure also the positive control. **S2B Fig**: Same as Fig S2A but longer exposure. S3C Fig: Filter 4 was process in a similar way but with the left part visualized with the mouse monoclonal antibody, which gave a strong band corresponding to the CYP2W1 in colon cancer sample and some additional unspecific bands. This antibody did not recognize any CYP2W1 in normal adrenal. → Indicates protein band corresponding to CYP2W1. **S2D Fig**: Filter 5 shows WB vizualized with the CYP2W1 mouse monoclonal sc-374426. 25 μg protein of 800 x g supernatant from one normal adrenal cortex, nADR125, and two ACC samples, C6 and the CYP2W1 containing Ca29. HEK-CYP2W1 as positive control and HEK-Mock as negative control.
(TIF)

S3 Fig Tissue blot (Cat no 1522 from BioCat GmbH, Heidelberg, Germany) and comparison of three different CYP2W1 antibodies, A Thermo Fisher Scientific PA5-14900, B Our C-term ‘852’, C SantaCruz monoclonal sc-374426. The blot (filter 6) was fist visualized with the mouse monoclonal antibody (antibody C), then with the Thermo Fisher antibody (antibody A)and after stripping the filter also with the 852 antibody (antibody B). The white bands in the background after the last staining are due to the color staining of the blot due to the heavy reaction with the Thermo Fisher Scientific antibody.
(TIF)

S1 Table Details of ACC samples included in the study. (DOCX)

S2 Table RT-PCR Ct-values. (XLSX)
