# Supplementary figures and images for: Human Cytochrome P450 2W1 Is Not Expressed in Adrenal Cortex and Is Only Rarely Expressed in Adrenocortical Carcinomas
Source: PLoS One. 2016 Sep 6;11(9):e0162379. doi: 10.1371/journal.pone.0162379 (PMC5012573; doi:10.1371/journal.pone.0162379)

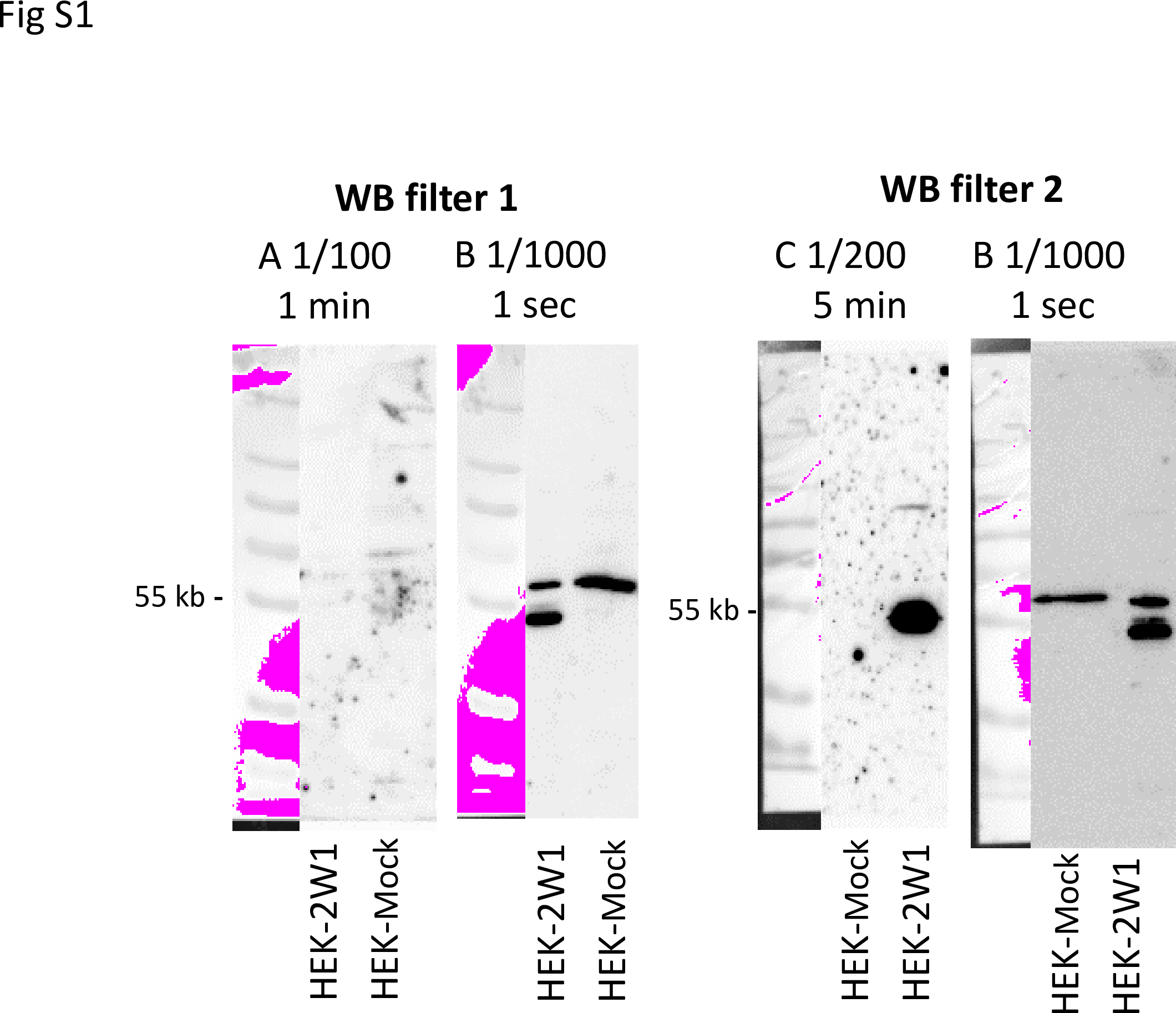

Supplement: S1 Fig — Comparison of three different CYP2W1 antibodies using WB filters with 800 x g supernatant from HEK293 cells expressing CYP2W1 and the corresponding cells without CYP2W1 expression (HEK-Mock). Antibodies used: A Thermo Fisher Scientific PA5-14900, B Our C-term ‘852’, C SantaCruz monoclonal sc-374426. WB filter 1 was first incubated with antibody A using goat anti-rabbit IgG-HRP for visulization and subsequently with antibody B using the same secondary antibody. WB filter 2 was fist incubated with antibody C using goat anti-mouse IgG-HRP for visualization and subsequently with antibody B using goat anti-rabbit IgG-HRP. The indicated time corresponf to exposure time. (TIF) [file pone.0162379.s001.tif]

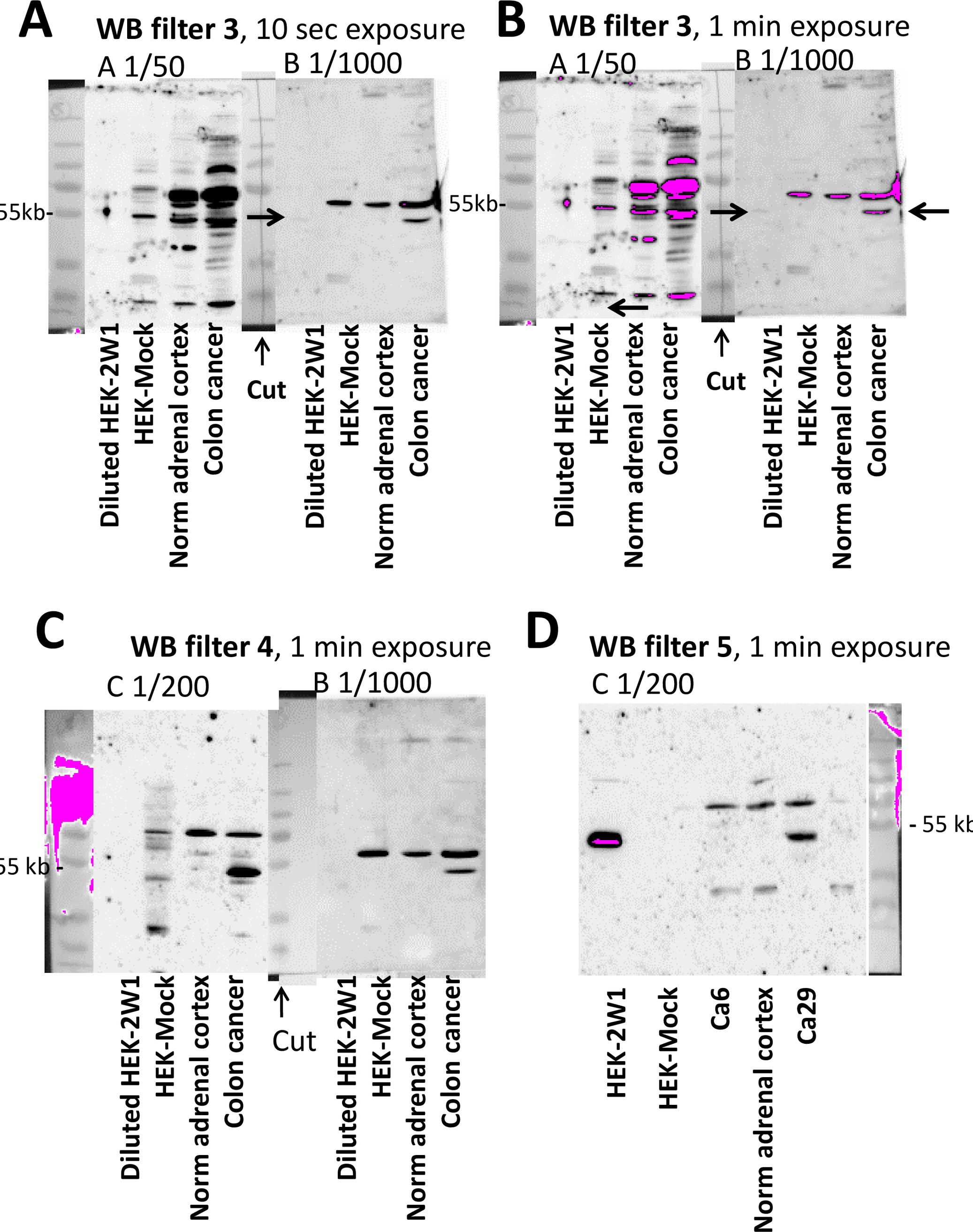

Supplement: S2 Fig — Thermo Fisher Scientific PA5-14900 antibody A, Our C-term ‘852’ (antibody B), C SantaCruz monoclonal sc-374426 (antibody C). Four identical WB filters were prepared with 800 x g supernatant from HEK293 cells expressing CYP2W1 (diluted sample) and the corresponding cells without CYP2W1 expression (HEK-Mock), normal adrenal cortex, nADR129, (25 μg protein) and a colon cancer sample with previously known expression of CYP2W1 (25 μg protein). S2A Fig: The left part of filter 3 was incubated with the Thermo Fisher antibody and the right part with our 852 antibody. The former generated a lot of bands also having mobilities similar to CYP2W1, whereas the 852 antibody visualized CYP2W1 only in the colon cancer sample and after longer exposure also the positive control. S2B Fig: Same as Fig S2A but longer exposure. S3C Fig: Filter 4 was process in a similar way but with the left part visualized with the mouse monoclonal antibody, which gave a strong band corresponding to the CYP2W1 in colon cancer sample and some additional unspecific bands. This antibody did not recognize any CYP2W1 in normal adrenal. → Indicates protein band corresponding to CYP2W1. S2D Fig: Filter 5 shows WB vizualized with the CYP2W1 mouse monoclonal sc-374426. 25 μg protein of 800 x g supernatant from one normal adrenal cortex, nADR125, and two ACC samples, C6 and the CYP2W1 containing Ca29. HEK-CYP2W1 as positive control and HEK-Mock as negative control. (TIF) [file pone.0162379.s002.tif]

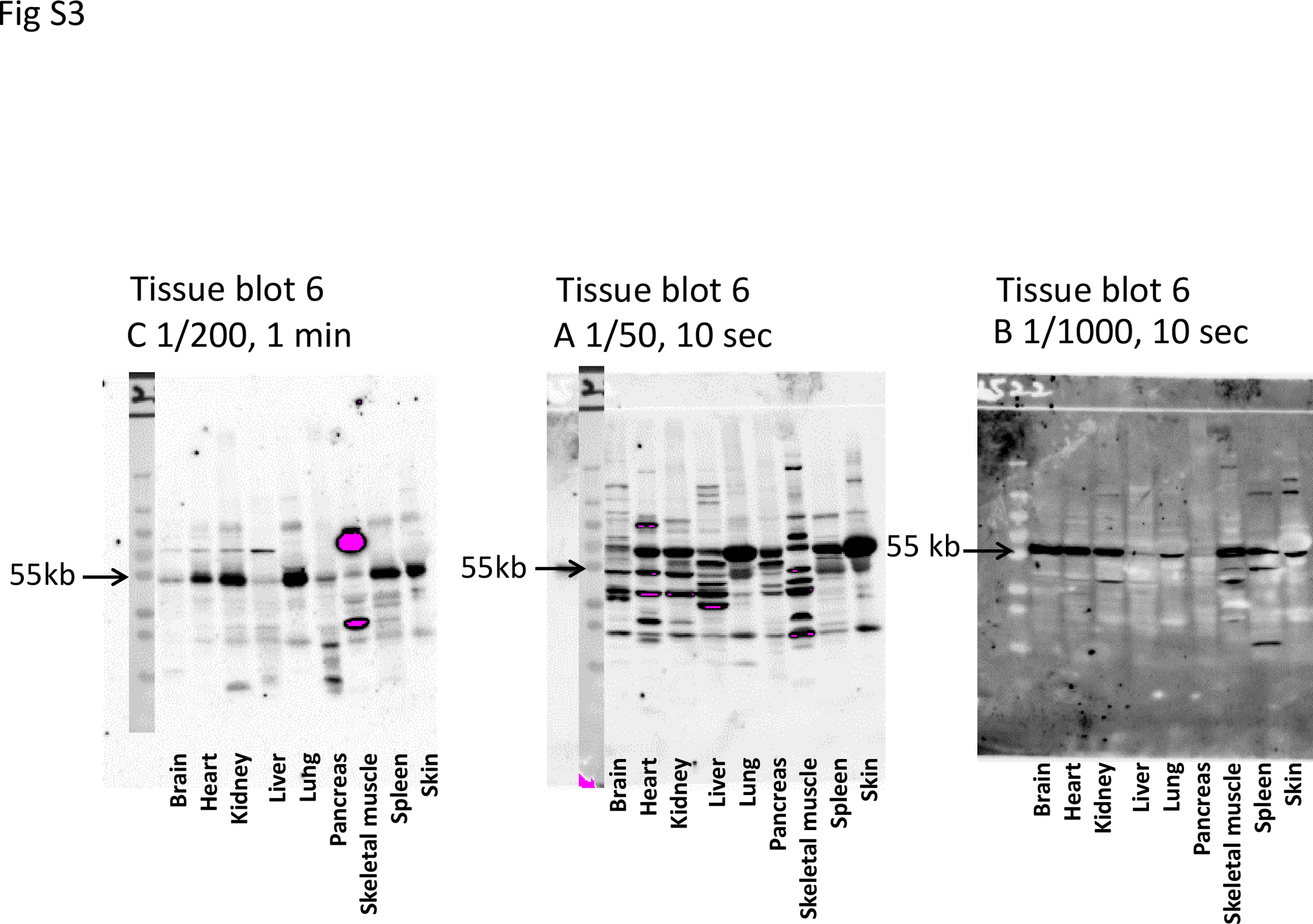

Supplement: S3 Fig — The blot (filter 6) was fist visualized with the mouse monoclonal antibody (antibody C), then with the Thermo Fisher antibody (antibody A)and after stripping the filter also with the 852 antibody (antibody B). The white bands in the background after the last staining are due to the color staining of the blot due to the heavy reaction with the Thermo Fisher Scientific antibody. (TIF) [file pone.0162379.s003.tif]
