# Supplementary material for: Human Cytochrome P450 2W1 Is Not Expressed in Adrenal Cortex and Is Only Rarely Expressed in Adrenocortical Carcinomas
Source: PLoS One. 2016 Sep 6;11(9):e0162379. doi: 10.1371/journal.pone.0162379 (PMC5012573; doi:10.1371/journal.pone.0162379)
Supplement: S1 Table — (DOCX) [file pone.0162379.s004.docx]

**S1 Table**

Details of ACC samples included in the study.

| Sample | Gender | Age  (y) | Tumor Size  (cm) | Tumor Weight  (gr) | ENSAT  Stage | Hormone  profile | Previously included in publication |
| --- | --- | --- | --- | --- | --- | --- | --- |
| Ca 1 | F | 63 | 20 | 1500 | IV | T |  |
| Ca 2 | M | 78 | 15 | 203 | III | n.a. |  |
| Ca 3 | F | 72 | 7 | 85 | III | n.a. | [[1](#_ENREF_1)] |
| Ca 5 | M | 72 | 11 | 1036 | II | - |  |
| Ca 6 | F | 40 | 18 | 2350 | II | T, A, D | [[1](#_ENREF_1)] |
| Ca 7 | F | 56 | 9 | 309 | III | C, A | [[1](#_ENREF_1)], [[11](#_ENREF_11)] |
| Ca 8 | F | 54 | 15 | 1339 | IV | C |  |
| Ca 9 | M | 68 | 15 | 1420 | II | - |  |
| Ca 11 | M | 68 | 12 | 660 | II | - | [[11](#_ENREF_11)] |
| Ca 12 | F | 84 | 19 | 1700 | III | - | [[1](#_ENREF_1)] |
| Ca 13 | M | 64 | 21 | N.A. | II | - |  |
| Ca 14 | M | 67 | 19 | N.A. | II | A | [[11](#_ENREF_11)] |
| Ca 15 | M | 77 | 11 | 449 | II | - | [[11](#_ENREF_11)] |
| Ca 16 | M | 69 | n.a. | 1100 | IV | C, A | [[11](#_ENREF_11)] |
| Ca 17 | F | 68 | 10 | N.A. | II | - | [[11](#_ENREF_11)] |
| Ca 18 | F | 28 | 21 | 2600 | II | C | [[11](#_ENREF_11)] |
| Ca 19 | F | 61 | 14 | 585 | II | C | [[11](#_ENREF_11)] |
| Ca 20 | M | 60 | 10 | 262 | n.a. | - |  |
| Ca 21 | F | 40 | 9.6 | N.A. | IV | n.a. |  |
| Ca 22 | F | 59 | 10 | 273 | II | C | [[11](#_ENREF_11)] |
| Ca 23 | F | 63 | 9 | 145 | IV | C | [[11](#_ENREF_11)] |
| Ca 24 | F | 54 | 20 | 4400 | II | C, A | [[11](#_ENREF_11)] |
| Ca 25 | M | 49 | 16 | 1.7 | IV | C | [[11](#_ENREF_11)] |
| Ca 26 | F | 68 | 12 | 437 | II | n.a. | [[11](#_ENREF_11)] |
| Ca 28 | M | 51 | 19 | 1670 | n.a. | n.a. |  |
| Ca 29 | F | 35 | 8 | 336 | II | T | [[11](#_ENREF_11)] |
| Ca 31 | F | 35 | 9 | 138 | n.a. | - |  |

n.a.= Not available, Y = years; gr = gram
-.= Non-hormone-producing tumor
T= testosterone-producing tumor
A= aldosterone-producing tumor
D= dehydroepiandosterone (DHEA)-producing tumor
C= cortisol-producing tumor
